# The Correlation of a 2D Volume-Referencing Endolymphatic-Hydrops Grading System With Extra-Tympanic Electrocochleography in Patients With Definite Ménière's Disease

**DOI:** 10.3389/fneur.2020.595038

**Published:** 2021-01-20

**Authors:** Baihui He, Fan Zhang, Hui Zheng, Xiayu Sun, Junmin Chen, Jianyong Chen, Yupeng Liu, Lu Wang, Wei Wang, Shuna Li, Jun Yang, Maoli Duan

**Affiliations:** ^1^Department of Otolaryngology Head and Neck Surgery, Xinhua Hospital, Shanghai Jiaotong University School of Medicine, Shanghai, China; ^2^Ear Institute, Shanghai Jiaotong University School of Medicine, Shanghai, China; ^3^Shanghai Key Laboratory of Translational Medicine on Ear and Nose Diseases, Shanghai, China; ^4^Department of Radiology, Xinhua Hospital, Shanghai Jiaotong University School of Medicine, Shanghai, China; ^5^Department of Otolaryngology Head and Neck and Neurotology and Audiology, Karolinska Institute, Karolinska University Hospital, Stockholm, Sweden; ^6^Department of Clinical Science, Intervention and Technology, Karolinska Institute, Stockholm, Sweden

**Keywords:** endolymphatic hydrops, magnetic resonance imaging, Ménière's disease, electrocochleography, diagnosis

## Abstract

**Background:** Although magnetic resonance imaging (MRI) of the membranous labyrinth and electrocochleography (ECochG) have been used to diagnose endolymphatic hydrops (ELH) in patients with Ménière's disease (MD), the relationship between imaging and ECochG is not well-documented.

**Objectives:** This study evaluates the ELH using 3D-FLAIR MRI and extra-tympanic ECochG (ET-ECochG) and correlates the results from 3D-FLAIR MRI to those from ET-ECochG.

**Materials and Methods:** 3D-FLAIR MRI images of 50 patients were assessed using a 2D volume-referencing grading system (VR scores, relative scores according to the known volumes of the cochlea, vestibule, and semicircular canals). Forty healthy subjects were included and compared to 51 definite MD ears of 50 patients while analyzing the ET-ECochG, which used a self-made bronze foil electrode. The amplitude ratio of the summating potential (SP) to the action potential (AP) (SP/AP) and the area ratio of SP to AP (Asp/Aap) were collected. Relative ELH grade scores were then correlated to ET-ECochG (SP/AP, Asp/Aap).

**Results:** The VR scores showed a better correlation (*r* = 0.88) with the pure tone average (PTA), disease duration, and vertigo frequency of MD than the Bernaerts scores (grading the cochlea and vestibule separately) (*r* = 0.22). The SP/AP and Asp/Aap of the unilateral MD patients were statistically comparable to those measured in contralateral ears and the results between the definite MD ears with healthy ears were statistically comparable (*p* < 0.05). In a ROC analysis Asp/Aap (area under curve, AUC 0.98) significantly (*p* = 0.01) outperformed SP/AP (AUC 0.91). The total score of ELH, vestibular ELH, and cochlear ELH were also correlated with SP/AP and Asp/Aap. The strongest correlation was found between the Asp/Aap and cochlear ELH (*r* = 0.60).

**Conclusion:** The 2D volume-referencing grading system was more meaningful than the Bernaerts scores. A correlation was found between ELH revealed by 3D-FLAIR MRI and the SP/AP of ET-ECochG in evaluating definite MD patients. The Asp/Aap appeared a more sensitive and reliable parameter than SP/AP for diagnosing the ELH of the membranous labyrinth.

## Introduction

Menière's disease (MD) is a multifactorial inner ear disorder characterized by fluctuating sensorineural hearing loss, spontaneous vertigo attacks, tinnitus, and aural fullness (1). The pathophysiology of MD is still unclear and may be related to a combination of genetic effect and environmental factors (1, 2), while the histopathological association of MD and endolymph hydrops (ELH) in the cochlear and the vestibular organs is definite (3, 4). The development and progress in magnetic resonance imaging (MRI) techniques has enabled the visualization of ELH separately in cochlea and vestibule using a 3.0 Tesla (T) scanner and a gadolinium-based contrast agent (5–7). Recently, volume quantification methods of the inner ear, especially the endolymph, have been introduced. However, these methods require specific scan sequences, 3D workstations (8–10), or probabilistic atlas (11) that require imaging processing capabilities and therefore may be difficult to establish in a clinical setting. Relatively, clinicians prefer a 2D semi-quantitative grading system that is easily and quickly applicable without having to invest further time or resources, for example, the grading standard stated by Nakashima (12). Additional 2D grading methods have been proposed to grade the ELH in the cochlea and vestibule. However, 2D ELH grading methods grade ELH in the cochlea and vestibule separately (13–15) without considering the volume ratio between cochlea and the vestibule and excluded the semicircular canals. The volume ratio and the semicircular canals should be taken into consideration to better represent the total ELH of inner ears. Therefore, an adjusted grading standard was used in this study, according to the literature about the volume ratio (10) to evaluate the ELH of MD patients.

The diagnosis of MD is based on clinical manifestations and the pure tone audiogram according to the 2015 consensus statement of the Barany Society (1). Other auditory and vestibular tests like electrocochleogram (ECochG), vestibular-evoked myogenic potential (VEMP), and glycerol test are applied to provide supportive information (2, 16). Besides, MRI can support the diagnosis of MD by identifying ELH (16). The high expense and complex procedure limit the wide usage of MRI among early diagnosed patients. Therefore, a low-cost non-MRI-dependent diagnostic proxy for ELH is needed. ECochG is a method of directly recording the electrical activity of the cochlea and the acoustic nerve in response to acoustic stimuli (17, 18). It has been recognized that ECochG is a valuable tool in the diagnosis of MD due to its sensitivity to the hydrops in the membranous labyrinth (18, 19). However, the diagnostic value of ECochG for MD diagnosis was under debate (18, 20). Extra-tympanic ECochG (ET-ECochG) is a non-invasive protocol using an external auditory canal electrode. An elevation of the amplitude ratio of the summating potential (SP) to the action potential (AP) (SP/AP) of ECochG has been used as an adjunct to diagnose MD (18). It is likely that the analysis of the area ratio of SP to AP (Asp/Aap) increases the sensitivity of the test (19). However, the relationship between ET-ECochG and the grading of ELH is not well-documented. The purpose of this study was to establish a 2D volume-referencing ELH grading system and further explore the relationship between the degree of ELH with ET-ECochG in definite MD patients.

## Materials and Methods

### Ethics

This study was approved by the Ethics Committee of Xinhua Hospital, Shanghai Jiaotong University School of Medicine (Approval Number: XHEC-D-2020-119). Written informed consent was obtained from each participant.

### Subjects

In this retrospective study, clinically diagnosed MD patients were enrolled between August 2018 and June 2020 at the Department of Otolaryngology-Head and Neck Surgery, Xinhua Hospital, Shanghai Jiaotong University School of Medicine. The inclusion criteria were as follows: (a) patients with a clinical diagnosis of the definite MD according to the 2015 consensus statement of the Barany Society (1); (b) complete medical history record with ET-ECochG and MRI. The exclusion criteria were as follows: (a) chronic otitis media or a history of other middle or inner ear diseases; (b) history of middle or inner ear surgery; (c) lesions of the central nervous system and the inner ear or cerebellopontine angle; (d) history of vasodilators, diuretics or intratympanic gentamicin or dexamethasone treatment in the previous 2 weeks; (e) patients with claustrophobia, pregnancy, or allergy to Gadolinium-based contrast agents. Fifty patients met the inclusion criteria (51 definite MD ears; 28 females and 22 males; age range = 27–75 years; mean age = 53 years, SD = 12.2 years). All of their clinical history and examination results were collected from the medical history system.

Forty healthy subjects (80 ears; 23 females and 17 males; age range = 24–42 years; medium age = 27; quartile: 25–29 years) served as healthy controls for the ET-ECochG test using the bronze foil electrodes. Inclusion criteria for healthy subjects were as follows: normal otoscopy, pure tone thresholds better than 25 dBnHL from 0.25 to 8 kHz, normal tympanometry.

### MRI

#### Intratympanic Gd Injection

Gadopentetate dimeglumine (Xudonghaipu Pharmaceutical Co. Ltd., Shanghai, China) was diluted eight-fold with saline (v/v = 1:7) and injected intra-tympanically (0.5 ml) through the inferior-posterior quadrant of the tympanic membrane bilaterally using a 1 ml syringe connected to a 23 G needle under the microscope. The subjects were then kept in the supine position for 60 min.

#### Acquisition of MRI

MRI was performed 24 h after the application of the contrast agent on a 3.0 Tesla MR scanner (Umr 770, united-imaging, Shanghai, China), using a 24-channel head coil. Three-dimensional heavily T2-weighted spectral attenuated inversion recovery (3D-T2-SPAIR, T2) and 3D T2 fluid-attenuated inversion recovery (3D-FLAIR) imaging were subsequently performed. The scan parameters for the 3D-FLAIR sequence were as follows: voxel size = 0.78 * 0.78 * 1.1 mm, scan time = 6 min and 11 s, time of repetition (TR) = 6500 ms, time of echo (TE) = 286.1 ms, time of inversion = 1950 ms, flip angle = 67°, echo train length = 160 points, slice thickness = 0.6 mm, field of view = 200 * 200 mm, and matrix size = 256 * 256. We used a heavily T2-weighted SPAIR sequence with the detailed scan parameters as follows: voxel size = 0.65 * 0.52 * 0.76 mm, scan time = 4 min and 30 s, TR = 1,300 ms, TE = 254.7 ms, flip angle = 110°, slice thickness = 0.4 mm, field of view = 200 * 200 mm, and matrix size = 384 * 384.

#### Image Processing of MRI

**Figure 1:**
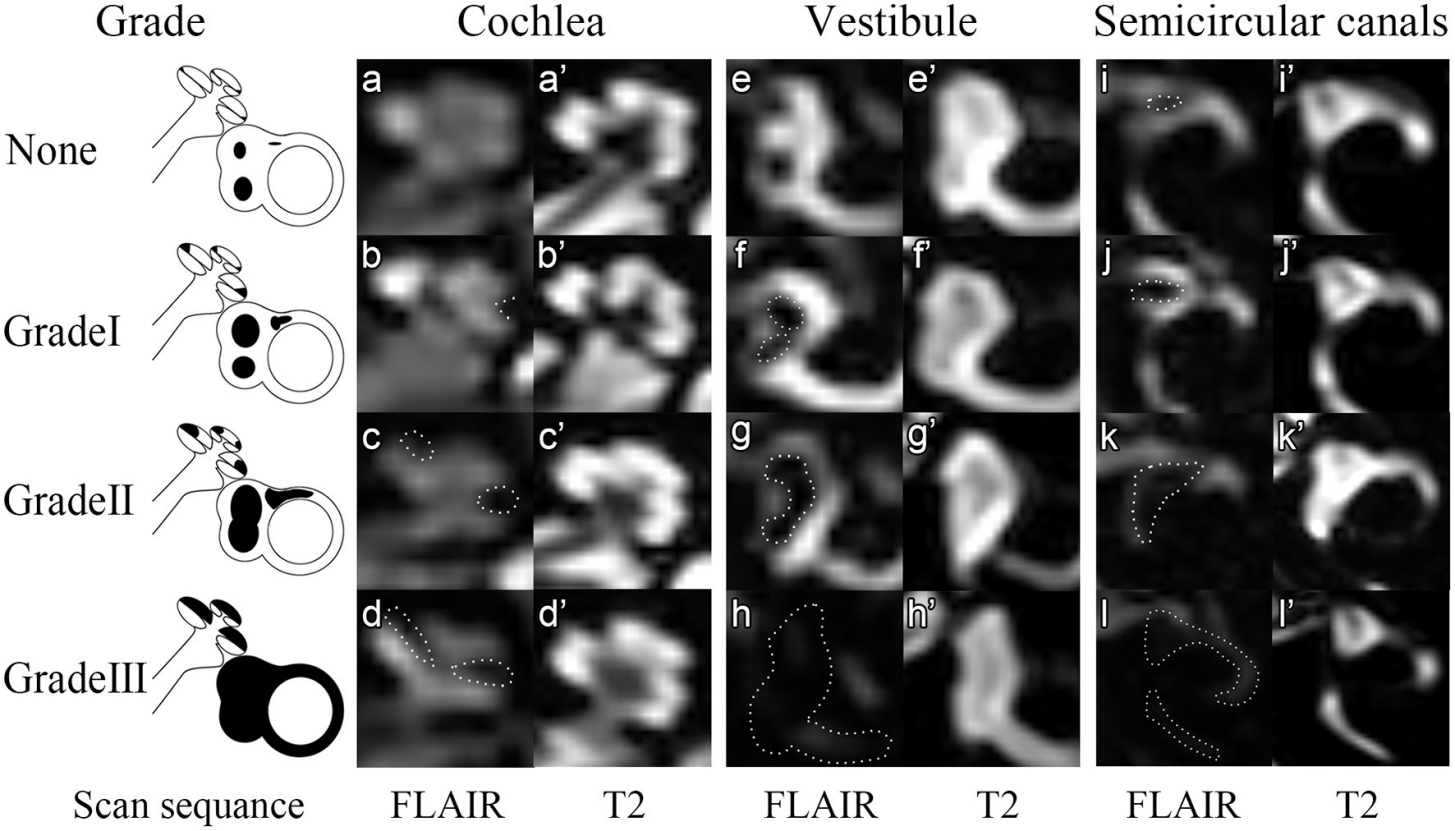
Four grades of the inner ear ELH. In this figure, 3D-FLAIR pictures **(a–l)** are shown adjacent to T2 weighted image for each grade **(a'–l')**. None = Normal inner ear. In the normal inner ear, one can recognize no enlargement in the scala media **(a,a')**. The saccule and utricle are separate and saccule is smaller than the utricle **(e,e')**. The semicircular canals and a narrow dark area (<1/3 of the ampulla) are visible **(i,i')**. Grade I = Mild hydrops. In mild hydrops cochlea, an enlarged scala media can be observed with the scala vestibuli around **(b,b')**. In mild hydrops vestibule, saccule becomes larger than utricle but not yet confluent with utricle **(f,f')**. In mild hydrops semicircular canals, a dark area occupying over 1/3 of the ampulla is observed **(j,j')**. Grade II = Moderate hydrops. In moderate hydrops cochlea, the scala media expands into circle **(c,c')**. In the vestibule, a confluence of the saccule and utricle appears with visible perilymph surrounding **(g,g')**. In semicircular canals, the crura become dark and some of the canals become invisible. Grade III = Severe hydrops. The scala vestibuli, saccule and utricle and semicircular canals are filled with endolymph so that no signals could be viewed in these areas.

The images were evaluated separately by two experienced radiologists with 7-year and 25-year working experience. The radiologists were blinded to the patient's information according to an adjusted 2D volume-referencing semi-quantification grading system (VR scores, Figure 1). We preferred 2D semi-quantification analysis and made an adjusted criteria according to the inner ear fluid volume (21) and the normal endolymph ratio (10) described by Inui et al. and Ito et al. (Table 1). In brief, the degree of ELH in the vestibule and cochlea was assessed by visual comparison of the relative areas of the non-enhanced endolymphatic space vs. the contrast-enhanced perilymph space in several axial planes. The maximum hydrops' volumes for the vestibule and semicircular canals of the inner ear were estimated as the maximum inner ear fluid's volume of each part. However, in the cochlea, the endolymphatic hydrops seldom herniated into the scala tympani due to the bony spiral plate according to the images previously published (12, 22, 23). The volume ratio of the scala tympani vs. scala vestibuli (including the scala media) according to the area data describe by Wysocki et al. (24) is about 1.04. Therefore, the estimated maximum hydrops volume for cochlear ELH accounts for about 1/(1 + 1.04) of the total cochlear inner ear fluid volume or approximately 55.6 μl. To mark the hydrops of each part of the inner ear, we defined the same expansile volume represent for 1 point in each part. Since three semicircular canals have five crura and three canals, we supposed each semicircular canal or crus had a similar volume according to the reported guinea pigs data (25) and human segmentation pictures showed by Inui et al. (21). We also supposed ELH was distributed evenly in each part for easier calculation for the volume scores, and each part was marked as 1 point if the perilymph became narrow (mild hydrops) and 2 points if it became dark (severe hydrops). Therefore, the maximum hydrops' score for semicircular canals was 16 points, which represented the maximum expansile volume of ELH in semicircular canals (78.2 μl, each point = 4.89 μl). Accordingly, the cochlea was counted 9 points considering the relative expand volume while the vestibule was counted 12 points for the maximum hydrops' scores (Table 1). Then we adopted the semi-quantification grading system described by Bernaerts et al. (13) to separate vestibular hydrops into four degrees (Bernaerts scores). However, as for the cochlear hydrops, the cochlear endolymphatic expand volume was more than twice the volume of the scala media according to the human cochlear anatomy showed by Raufer et al. (26) and according to the calculated results (Table 1). So we separated the hydrops of cochlea into four degrees (Figure 1-cochlea) by adding a degree between normal cochlea and Grade I described by Bernaerts et al. because we discovered that for some patients the scala media could expand a bit without forming a round dark circle (Figure 1b). No grading system was proposed for semicircular canals before this study because researchers seldom viewed hydrops in semicircular canals. However, some reported vestibular herniation into the crura of the semicircular canals (23, 27). Among these studies, Sugimoto et al. defined that visible black area exceeded 1/3 or more as ELH herniation in the lateral and posterior semicircular canals. Furthermore, a small dark area can be found in asymptomatic contralateral ears frequently (Figure 1i). Given the convenient 2D semi-quantification in both vestibule and cochlea, the grading for semicircular canals was simplified by mainly evaluating and separating the hydrops in the lateral semicircular canal into four degrees. Therefore, we defined a small visible herniation which was 1/3 less than the semicircular canal with perilymph surrounding as no hydrops (None), a larger herniation (>1/3) with hydrops as Grade I and the total invisibility of crura, which often accompany the stenosis of canals as Grade II. If all semicircular canals were invisible, we defined as Grade III (Figure 1-semicircular canals). Finally, we analyzed the scores of each part of the inner ear separately (Table 1) and also in sum with the clinical history and ET-ECochG results. As reported in our previous study, patients with a long history of MD and severe hearing loss in the definite MD ears are more likely to exhibit endolymphatic hydrops in the asymptomatic contralateral ear (28), we thereby excluded asymptomatic contralateral ears of patients with unilateral MD and only include ears with definite MD. The ELH of each definite MD ear was assessed by both the VR scores and the Bernaerts scores. We calculated the sum scores of the ELH to provide a semi-quantitative assessment of total inner ear hydrops. The Sum score of the ELH of a definite MD ear equaled the sum of the score of the cochlea, the score of the vestibule, and the score of the semicircular canals according to our VR scores. The sum score of the Bernaerts scores included only the sum of the grade of the cochlea and vestibule. We also calculated the sum score of the vestibule with the semicircular canals for the herniation hypothesis and presented this sum as the entire vestibular ELH.

**Table 1:** Relative maximum volume referencing scores (VR scores) of different parts in the inner ear and the relative points of four gradings of each part.

**Content**	**Cochlea**	**Vestibule**	**Semicircular canal**
Volume of inner ear fluid (μl) (21)^*^	113.5	69.0	91.8
ELH space (%) (10)	10.2	17.7	14.8
ELH volume (μl) = Volume of inner ear fluid^*^ ELH space	11.6	12.2	13.6
Hydrops volume_max_ (μl)^**^	55.6	69.0	91.8
Expansile volume_max_ (μl)^***^ = Hydrops volume_max_ – ELH volume	44.0	56.8	78.2
Relative hydrops scores (points)	9	12	16
GRADINGS OF ELH^****^			
None: no hydrops	0	0	0
GradeI: mild hydrops	3	4	5
GradeII: medium hydrops	6	8	10
GradeIII: severe hydrops	9	12	16

* *Inner ear volume: The calculated inner ear volume for different parts of the human inner ear, taken from Inui et al. (21) as the mean of bilateral ears in both genders*.

** *Hydrops volume_max_: The maximum volume for endolymphatic hydrops, calculated according to the percentage showed by Ito et al. (7). In the cochlea, hydrops seldom herniates into the scala tympani, thus the volume of scala vestibuli was used as the maximum volume*.

*** *Expansile volume_max_: The estimated maximum volume for maximum endolymph expansion of each parts of the inner ear*.

**** *Gradings of ELH: The scores of each part of the inner ear were rounded to the nearest integer according to the relative expand volume of the semicircular canals which contained 16 points. Sixteen came from the assumptions that three semicircular canals and five crura had similar volume and ELH was distributed evenly in each part, in which we marked 1 point if the perilymph became narrow (mild hydrops) and 2 points if became dark (severe hydrops). Then each part had four points for four grades which were divided equally and rounded to integers. However, for Grade II of the semicircular canals, we rounded the points down to 10 rather than 11 because we noticed that most canals and crura were narrow but still visible compared to the severe hydrops' ones*.

### ET-ECochG

#### Recording Procedure of ECochG

Bio-logic® Navigator Pro auditory evoked system 7.0.0 (Natus Medical Inc., San Carlos, CA, USA) was used for electrocochleography. The extra-tympanic electrode connecting a bronze foil was self-made. The bronze foil was chosen due to its availability and lower price than the gold one in order to reduce the cost of routine clinical expenses. Besides, bronze has better electric conductivity, which may improve the acquisition of small signals of the ET electrode compared with the tympanic electrode (29). Furthermore, the placement of a TM electrode on the tympanic membrane must be completed under an otoscope, which is a little difficult and time-consuming for audiologists. ET electrode could reduce the discomfort and made this test more acceptable for patients as well. The reference electrode was placed on the mastoid of the non-tested ear, whereas the ground electrode was placed on the forehead. Prior to electrode placement, all electrode sites were treated with an alcohol wipe and a lightly abrasive skin prepping gel using a cotton swab to ensure maximum adhesion and conductivity. Prior to placing the earphone into the ear canal, the ET electrode was covered with the Ten 20 conductive electrode paste and then surrounding the ER-3A insert earphone. The impedance of the ET electrode was kept below 5 kΩ. The other electrode impedances were kept below 1 kΩ. Alternating click stimuli of 100 μs duration at a rate of 11.3/s at 90 dBnHL of hearing level were presented monaurally. Intensity delay of 0.8 ms and the filter setting of 0.1–3 kHz were used. For ensuring the good quality of the responses, the stimuli were presented with 2,000 sweeps. For adequate visualization, the acquired responses were amplified 100,000 times. The SP and AP data were obtained, and each waveform was replicated at least two times for each subject. During the testing, the examinee lay comfortably on the bed in a soundproof room in the Diagnosis and Treatment Center of Hearing Impairment and Vertigo in Xinhua Hospital.

#### Measurement of the Amplitude Ratio and Area Ratio

**Figure 2:**
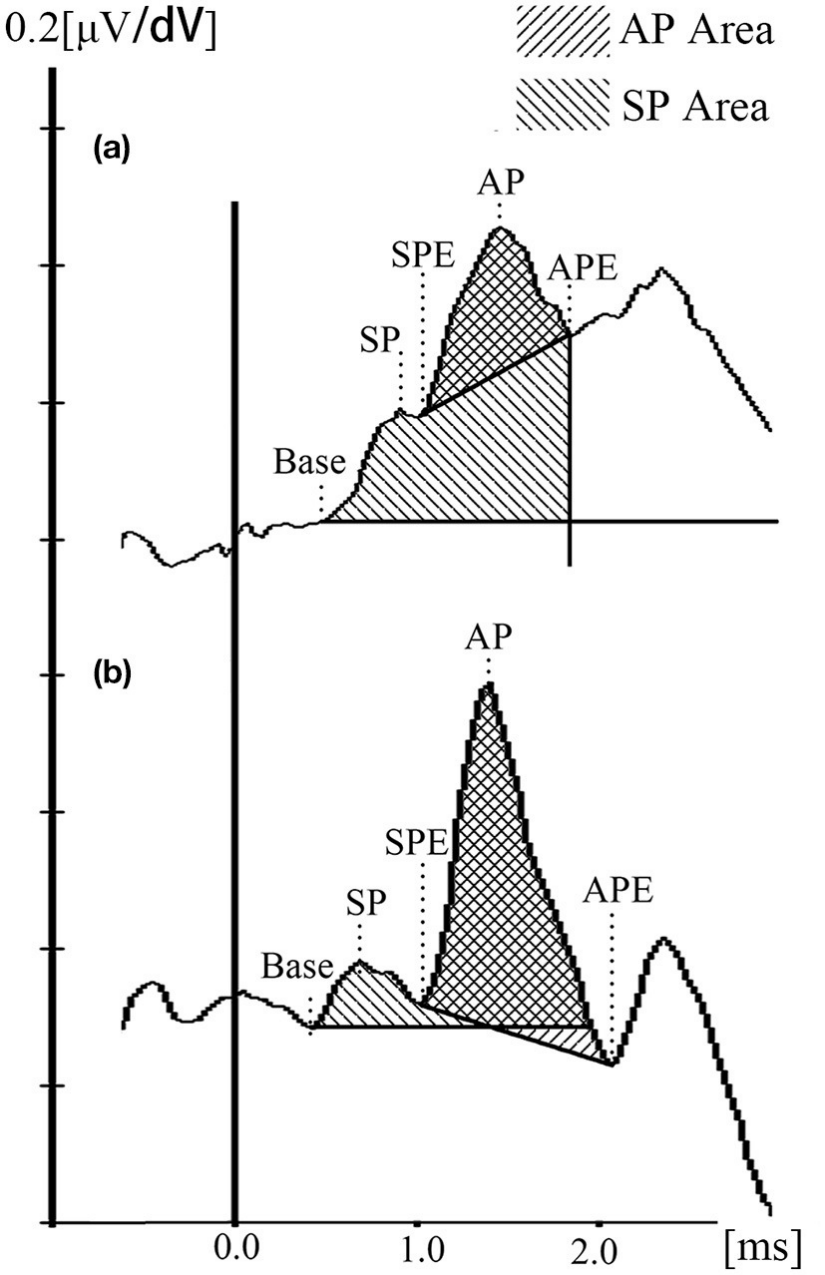
ET-ECochG waveforms and the calculation method. **(a)** Waveform of an definite MD ear. **(b)** Waveform of a healthy ear. SP duration = SPE — Base; AP duration = APE — SPE.

In the current study, the Auditory Evoked Potential system attached to the Bio-logic system was used to analyze first, and the waveforms were inverted before analyzed. For further figure analysis, such as area calculation, photoshop CC 2018 (Adobe, San Jose, CA, USA) and Image J 1.52 p (NIH, USA) were used. The baseline was defined as a horizontal line crossing the base point, which was the onset of the initial peak of SP of the waveform (determined by at least two repeat tracings) (30). The AP amplitude was determined from the baseline to the first large peak (represent the N1 of AP) between 1.0 and 2.0 ms after the SP peak. The latencies of the SP, AP, and their duration were collected. It has been reported that the SP/AP area ratio could improve the sensitivity and specificity of MD diagnosis (31, 32). However, this conclusion was argued by later researches (33, 34). Ferraro et al. firstly mentioned the area ratio because a widening of the SP/AP complex in patients with MD and the notice of the trailing edge of the AP-N1 does not return to baseline (35). Ferraro and Devaiah et al. thereby defined the SP area as the space under the curve from baseline to the next point where the waveform returned to baseline, and the AP area was defined from the onset of the AP to its first negative peak following N1 (32, 35). However, we found that few MD patients ECochG waveforms returned to the baseline, and the SP area would be infinite (Figure 2A). Therefore, we set a vertical line crossing the endpoint of N1 to make the SP area countable for those waveforms. The different methods and measurement standards might result in various debates for the clinical meaning of the ECochG and normal values for each center was recommended (36). Due to the new calculating method and the bronze foil ET electrode used in the current study, we collected 40 healthy subjects to establish the normative data for SP/AP amplitude ratio and area ratio (Figure 2B).

### Pure-Tone Audiometry

Pure-tone audiometry was performed in a soundproof room with the use of an audiometer (Type Madsen, Astera, Denmark). The Pure-tone average (PTA) was calculated as the mean of 0.5, 1 k, 2 k, and 4 k Hz air conduction thresholds (37, 38). Stage criteria were not mentioned in the 2015 consensus, so the clinical MD stage was classified according to the 1995 AAO-HNS guidelines by dividing PTA into four stages: I 0–25 dBnHL, II 26–40 dBnHL, III 41–70 dBnHL, IV > 70 dBnHL.

### Statistical Analysis

The data were analyzed using SPSS 26.0 for Windows (IBM, Chicago, IL, USA). Descriptive statistics were done for age, gender, clinical MD stage, disease duration (years), and vertigo attack frequency (vertigo episodes per month) in MD patients. The normal distribution test was done before the analysis of continuous variables. The paired *t*-test and independent *t*-test were used to analyze the differences between normatively distributed groups. The Mann-Whitney U-test was used for non-normal data. As for degrees of ELH and its correlation with clinical features (PTA, clinical course, vertigo frequency), the Pearson, Spearman, and Kendall correlation coefficient were used for different paired variables. The ordinal variables like the ELH points were analyzed again with the graded variables of all clinical features. Each variable was calculated several times in the same correlation comparison, so the Bonferroni correction was done. Furthermore, the canonical correlation was done for the correction of the normative linear data. To interpret correlation coefficients Cohen's standard classification ranges were used (weak 0.10–0.29, moderate 0.30–0.49, strong ≥ 0.50, Jacob Cohen, 1987). F test was used for the normative distributed data among several groups. ROC curve was analyzed using SPSS and Medcalc 19.3.1 (MedCalc Software Ltd., Ostend, Belgium) for the area under the curve (AUC), best cut-point, and difference analysis. The difference was considered to be statistically significant when *p* < 0.05.

## Results

### Clinical Characteristic of MD Patients

Fifty patients were enrolled in this study, 28 females (56%) and 22 males (44%), with a mean age of 53 ± 12.2 years. All patients had unilateral MD except one who had bilateral MD (51 definite MD ears). All patients presented with episodic vertigo (at least more than twice) and fluctuating low-frequency sensorineural hearing loss. Each vertigo attack lasted 20 min−12 h. The distribution of clinical stages of MD, disease duration, and frequency of vertigo episodes per month were shown in Table 2. Tinnitus was reported more frequently than ear fullness in definite MD ears in these patients.

**Table 2:** Clinical characteristic of definite MD ears.

**Characteristic**	**Number of ears (%, *n* = 51)**
**GENDER**	
Male	22 (43.14)
Female	29 (56.86)
**SIDE**	
Left	22 (43.14)
Right	29 (56.86)
**CLINICAL STAGE**	
I	5 (9.80)
II	11 (21.57)
III	29 (56.86)
IV	6 (11.76)
Tinnitus	49 (96.08)
Ear fullness	44 (86.27)
DISEASE DURATION^*^	
D1: <1 year	10 (19.61)
D2: 1–3 years	14 (27.45)
D3: 3-6 years	14 (27.45)
D4: >= 6 years	13 (25.49)
VERTIGO FREQUENCY (IN LAST MONTH)^**^	
V1: <1 time (<1 per month)	11 (21.57)
V2: 1–4 times (<1 per week)	13 (25.49)
V3: 4–15 times (<1 every 2 days)	7 (13.73)
V4: >= 15 times (>1 every 2 days)	15 (29.41)
**GRADING OF COCHLEA (POINTS)**	
None (0)	1 (1.96)
Grade I (3)	17 (33.33)
Grade II (6)	27 (52.94)
Grade III (9)	6 (11.76)
**GRADING OF VESTIBULE (POINTS)**	
None (0)	0 (0.00)
Grade I (4)	15 (29.41)
Grade II (8)	23 (45.10)
Grade III (12)	13 (25.49)
**GRADING OF SEMICIRCULAR CANALS (POINTS)**	
None (0)	35 (68.63)
Grade I (5)	12 (23.53)
Grade II (10)	3 (5.89)
Grade III (16)	1 (1.96)

* *Disease duration: the total disease duration from the onset of vertigo. The four levels (D1–D4) were divided according to the quartiles of our data*.

** *Vertigo frequency (in last month): the times of vertigo in the last month according to the recorded history. The four levels (V1–V4) were divided according to the quartiles of our data*.

### ELH Evaluation and MRI Association With Clinical Characteristics

**Figure 3:**
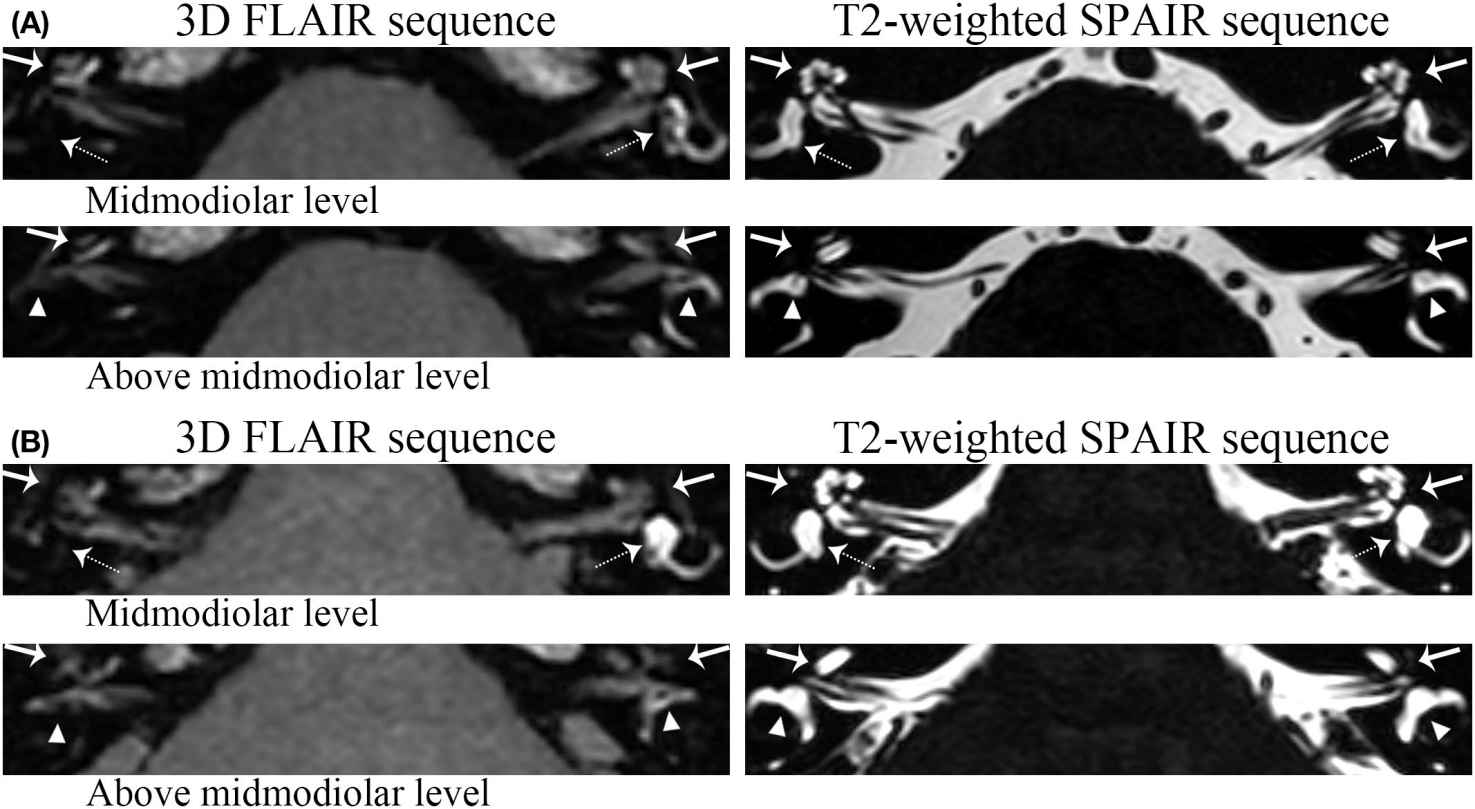
Grading examples of the ELH. **(a)** Patient I. For this patient, volume-referencing scores for each ear was marked as follows: Left ear: cochlea (arrow)-0, vestibule (dashed arrow)-0, semicircular canals (triangle) (Figure 1i)-0; Right ear: cochlea-9; vestibule-12; semicircular canals: 16. **(b)** Patient II: These pictures belong to the bilateral MD patient involved. Volume-referencing scores for each ear was marked as follows: Left ear: cochlea-3, vestibule-0, semicircular canals-5; Right ear: cochlea-6; vestibule-12; semicircular canals: 10. The right semicircular canals were graded 10 because some parts of the canals were still visible.

ELH was detected in 51 definite MD ears (100%) and 19 asymptomatic contralateral ears (38.76%) of the 49 unilateral MD patients. No adverse effects or complications were observed after the intratympanic administration of gadolinium. The degree of ELH evaluated by the VR scores was found to have different degrees of ELH in the vestibule, cochlea, or semicircular canals shown in 3D-FLAIR MR images (e.g., Figure 3). Cochlear ELH and vestibular ELH were more detectable than ELH in semicircular canals in most patients (Table 2). Spearman correlation analysis showed the cochlear ELH was correlated with the vestibular ELH (*r*_*s*_ = 0.44, *p* < 0.01) and the entire vestibular ELH (*r*_*s*_ = 0.43, *p* < 0.01). The sum score of the total inner ear ELH in different clinical stages was revealed significantly different by the *F*-test (*F* = 7.81, *p* < 0.01). There was no correlation between vestibular ELH and the semicircular canals' ELH. The individual multiple correlation analysis revealed the significant correlation between cochlear ELH with the disease duration (*r*_*s*_ = 0.35, *p* = 0.01) and the vertigo frequency (*r*_*s*_ = 0.36, *p* = 0.01) (*p* < 0.017). The sum ELH of VR system correlated significantly with the disease duration (*r*_*s*_ = 0.47, *p* < 0.01). However, no statistically sufficient relationship was shown between the PTA (*r*_*s*_ = 0.20, *p* = 0.15) and the vertigo frequency (*r*_*s*_ = 0.29, *p* = 0.04) with the sum ELH (*p* < 0.017). The difference was considered to be statistically significant when *p* < 0.017 for this part according to Bonferroni correction because all the data were cited three times for analysis. To better compare the VR scores with the Bernaerts scores, the adjusted Canonical correlation was calculated between the clinical features (include PTA, disease duration, and vertigo frequency) and the ELH (include the VR scores and the Bernaerts scores) to reduce the possible errors caused by multiple comparisons. The correlation of the VR sum scores was closer with all clinical features (*r* = 0.88) than the Bernaerts sum scores (*r* = 0.22) according to the correlation formula between clinical features to two different scores (Table 3-**u1**). Disease duration presented a closer correlation (*r* = 0.74) to the ELH (Table 3**-v1**).

**Table 3:** The correlation of ELH with clinical features by the two different grading systems.

**Contents**	Sum VR^*^	**Sum B**	**S_**v**_**	**S_**c**_**
PTA [rs(*p*)^**^]	0.20 (0.15)	0.51 (<0.01)	0.20 (0.15)	0.15 (0.30)
Disease duration (DD)	0.47 (<0.01)	0.12 (0.39)	0.47 (<0.01)	0.35 (0.01)
Vertigo frequency (VF)	0.29 (0.04)	0.14 (0.32)	0.22 (0.12)	0.36 (0.01)
Clinical stage	0.17 (0.24)	0.51 (<0.01)	0.17 (0.24)	0.12 (0.40)
D1–D4	0.44 (<0.01)	0.07 (0.61)	0.35 (<0.01)	0.25 (0.05)
V1–V4	0.25 (0.07)	0.02 (0.90)	0.16 (<0.01)	0.31 (0.01)
St ANDARDIZED CANONICAL CORRELATION COEFFICIENTS^***^				
u1 = −0.88 ^*^ Sum VR −0.22 ^*^ Sum B				
v1 = −0.73 ^*^ DD −0.44 ^*^ ln(PTA) −0.29 ^*^ VF				

* *Sum VR: the total ELH score (sum of three inner ear parts) according to the VR scores; Sum B: the sum of grades of cochlea and vestibule according to the Bernaerts scores; S_V_: the score of ELH in vestibule according to the VR scores; Sc: the score of ELH in cochlea according to the VR scores*.

** *r_S_ (p): Results were presented as coefficient of the spearman analysis with the p-value. Bonferroni's correction for multiple correlations: p < 0.017*.

*** *The normative linear variables were calculated, in which ln(PTA) were used*.

### ET-ECochG Analysis

The results of ET-ECochG were calculated in all definite MD ears (51 ears) and the asymptomatic contralateral ears (49 ears) in these MD patients. Given that the possibility of MD development of the asymptomatic contralateral ears, ECochG waveforms were also collected and calculated in 40 healthy subjects (80 ears) to make a contrast and better evaluate the diagnosis value. Forty-three of fifty-one definite MD ears (84.31%) had recordable ECochG waveforms. The 8 ears with unrecordable ECochG waveforms were composed of 1 Stage II MD ear, 6 Stage III MD ears, and 1 Stage IV MD ear (clinical MD stages according to PTA). Among patients with unrecordable ECochG, auditory thresholds were elevated in the high frequencies (68.75 ± 13.82 dB HL at 4 kHz; 78.13 ± 17.31 dB HL at 8 kHz). Affected and unaffected ears were first compared for the 49 unilateral MD patients. The paired-sample *t*-test revealed a significant difference of the SP/AP ratio (SP/AP) between the definite MD ears and the contralateral ears (*t* = 7.91, *p* < 0.01) (Table 4). There was a significant difference between the area ratio of SP/AP (Asp/Aap) of the definite MD ears and the contralateral ears by the Wilcoxon Man-Whitney-test (*p* < 0.01). The SP duration of the definite MD ears varied significantly from the contralateral ears (*p* < 0.01). AP latencies of definite MD ears were significantly longer than the contralateral ears (*p* = 0.04).

**Table 4:** Significantly varied data of the ECochG in unilateral MD patients.

	**SP/AP**		**Sp duration (ms)**	
	**Symptomatic ears**	**Contralateral ears**	**Symptomatic ears**	**Contralateral ears**
Mean ± SD	0.49 ± 0.16	0.28 ± 0.16	0.66 ± 0.18	0.54 ± 0.14
*t*-value	7.91		3.84	
*p*-value	<0.01		<0.01	
	**Asp/Aap**		**Ap latency (ms)**	
Quartile	3.12, 5.39	1.55, 2.38	1.52, 1.73	1.47, 1.66
*p*-value	<0.01		0.04	

**Figure 4:**
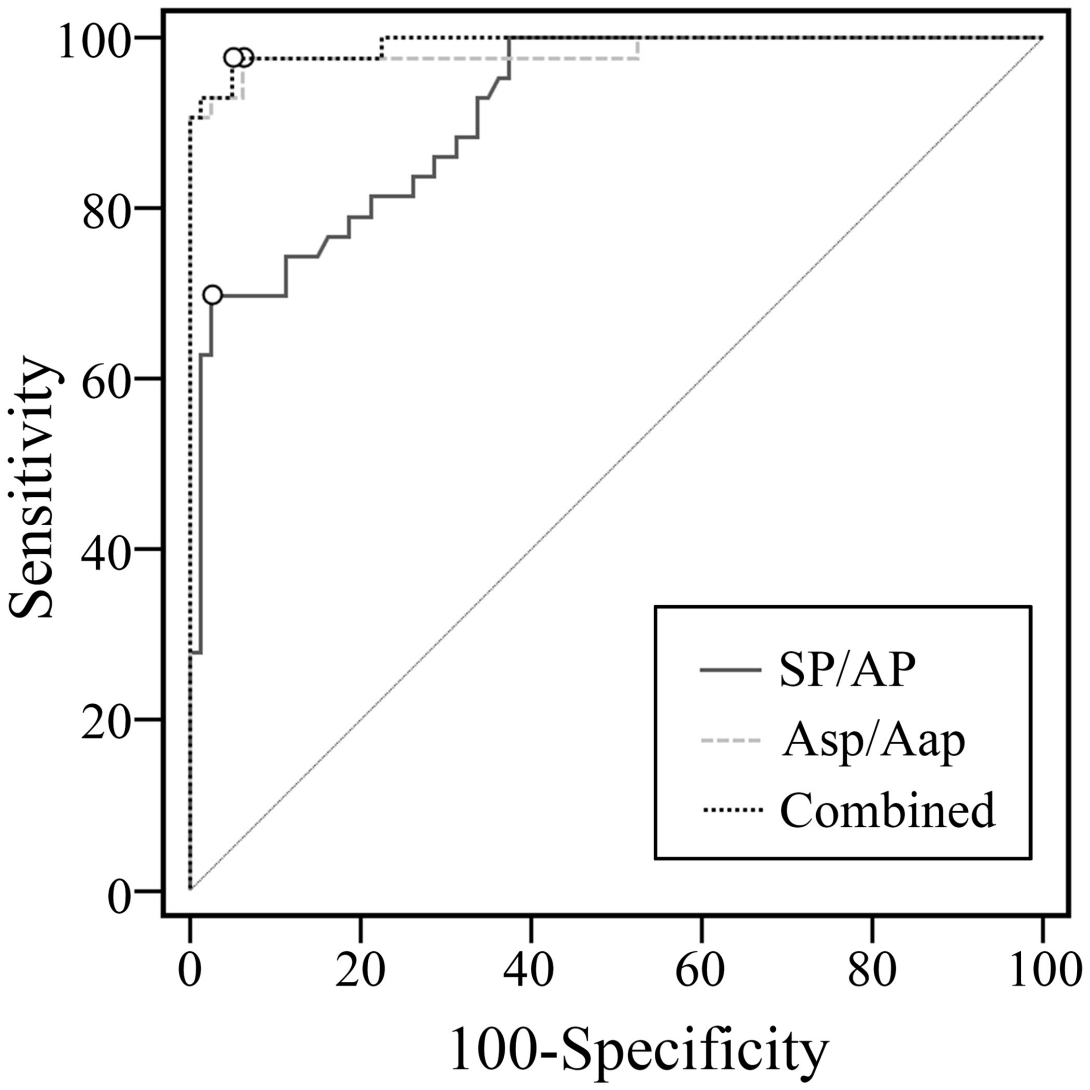
ROC curves of SP/AP, Asp/Aap and the two indexes combined.

For setting a diagnostic point for the ET-ECochG using bronze foil electrode, all definite MD ears (51 ears) and healthy ears (80 ears) were included for the ROC curves. We also drew the ROC curve for the combined assessment of two methods (Figure 4). The AUC of ROC curves can intuitively display the efficiency of the test index. The AUC of the Asp/Aap was 0.98, which was larger than the AUC of SP/AP (0.91). There was a significant difference of the ROC AUC between SP/AP with Asp/Aap (*p* = 0.01) and the combined assessment (*p* < 0.01) (Table 5). The cutoff point value of SP/AP was 0.38 (sensitivity = 69.77%, specificity = 97.50%), and the cutoff point value of Asp/Aap was 2.41 (sensitivity = 97.67%, specificity = 93.75%).

**Table 5:** Roc curve index and analysis.

**Contents**	**SP/AP**	**Asp/Aap**	**Combined**
Cut off value	0.38	2.41	*P* = 0.20
AUC	0.91	0.98	0.99
95%CI	0.85–0.96	0.94–1.00	0.96–1.00
*P*-value (vs. SP/AP)	/	0.01	<0.01

### Correlation Between ET-ECochG and MRI

Pearson or spearman analysis was done between the results of ET-ECochG and MRI of the definite MD ears (Table 6). Both SP/AP and Asp/Aap were significantly correlated with cochlear and vestibular ELH, the sum score of vestibular ELH, and the total score of inner ear ELH after the Bonferroni correction was done.

**Table 6:** Correlative analysis between ECochG and MRI.

**Indicaters**	Sv [r_**s**_ (p)^*^]	**Sc**	Sv + scc^**^	**Sum VR**
SP/AP	0.46 (<0.01)	0.41 (0.01)	0.46 (<0.01)	0.51 (<0.01)
Asp/Aap	0.41 (<0.01)	0.60 (<0.01)^*^	0.46 (<0.01)	0.55 (<0.01)

* *Asp/Aap had largest coefficient with cochlear ELH*.

** *Sv + scc: the sum score of vestibular and semicircular canals ELH. Bonferroni correction for multiple correlations: p < 0.0125*.

## Discussion

ELH, an excessive accumulation of endolymph in the cochlea (which is involved in hearing) and the vestibule (which is involved in balance), is a characteristic feature often observed in MD patients (2, 16). Foster et al. reported that MD patients had a near-certain probability of having endolymphatic hydrops in at least one ear (4). The relationship between the degree of ELH and clinical features was proved by researchers. Cochlear or vestibular ELH was correlated with the low-frequency hearing threshold according to Shi et al. (39). Yang et al. demonstrated a significant correlation between hearing loss and both the cochlear ELH and vestibular ELH (40). Bernaerts et al. reported a correlation between disease duration and the ELH of cochlea and vestibule (13). Gurkov et al. showed a correlation between cochlear ELH with both hearing loss and disease duration (23). Recently, the volumetric quantification was proposed to establish a methodological basis for further investigations into the pathophysiology and therapeutic monitoring of Menière's disease (8). The evaluation of the ELH volume required 3D volume analysis (9, 11). However, these 3D analyses are time intensive and impractical for routine work. Therefore, we proposed a convenient semi-quantification volume-referencing grading system according to the reported inner ear fluid volume results (9, 10). The VR scores showed a better correlation with the MD clinical features than the Bernaerts scores described by Bernaerts et al. (13). Thus, we believed that the sum score of hydrops of the entire inner ear according to the volume-referencing grading system had more diagnostic value than the Bernaerts scores, which graded the vestibule and cochlea separately.

MR imaging demonstrating inner ear fluid following intratympanic injection was reported in our previous research in 2004 (41) in guinea pigs and was first proposed in MD patients in 2007 (42). The first semi-quantification grading of ELH was raised in 2009 (12). Various improvements in MR protocols have been made afterward for higher quality images (43–46). However, the ELH grading system remained at the original level, which only evaluated the cochlea and vestibule without considering the relative volume. We referred to the four-grade semi-quantification vestibular grading system and the cochlear grading system mentioned by Bernaerts et al. (13) while analyzing. Gurkov et al. evaluated the cochlea into four grades but did not include the definition of how to divide four grades (23). Yang et al. then described the definition (40). Kirsch et al. (47) and Van et al. (48) then evaluated ELH of the total inner ear into four grades. We graded each part of the inner ear separately and differed from what Yang et al. mentioned. So we explained our grading carefully in the method part and showed in Figure 1. Since the pressure of perilymph and endolymph changed a little after ELH, according to the previous researches (49, 50), the total endolymph volume of the inner ear is much more important as measurement. Therefore, we recommend the volume-referencing grading system proposed in this study as it could easily and precisely estimate the ELH for the MRI images.

On the other hand, researchers hoped to explore more value of auditory and vestibular examinations, which would be easier for outpatients and provide diagnostic support for MD. This was because we observed that the high expense and complex procedure limited the wide usage of MRI among early diagnosed patients. ECochG was reported as a valuable tool in the diagnosis of MD due to its sensitivity to the ELH (18, 19). Cho et al. reported that the vestibular ELH was larger in patients with abnormal SP/AP than those with normal results (51). Yang et al. showed a correlation between SP/AP with vestibular ELH, but not with cochlear ELH (40). Our results revealed that ECochG was a valuable test for its correlation with the degree of ELH. Furthermore, the Asp/Aap had the largest coefficient to the cochlear ELH. The results supported the theory that ECochG reveals the potentials derived from the cochlea (17). It has been suggested that AP is derived from the afferent fibers in the cochlear nerve, and SP is a direct current potential arising in response to a current alternative stimulus (17). Our results showed that the duration of AP (nerve fibers) was not influenced by ELH while the duration of SP (summating response) was larger and had a high coefficient with the degree of ELH in the cochlea where the main response occurred. The underlying hypothesis of the enlarged SP is that hydrops-related inflation of the scala media leads to a static bias of the resting position of the organ of Corti and of outer-hair-cell stereocilia bundles and modified the electrical and mechanical properties of the cells (52).

However, according to the literature published, the diagnostic value of ECochG has been controversial (53) and some suggested that it had limited value as a clinical tool in the diagnosis of MD (34). The different reports might be due to the varied diagnostic value of ECochG, different electrodes, and multiple settings, which might influence the sensitivity and specificity of ECochG. Take SP/AP as an example, Quatre et al. (54) took >0.43 as abnormal, while Lamounier et al. (55) took >0.5. The absence of a shared ECochG procedure (electrode, stimuli type, and rate) made a great difference between the SP/AP and Asp/Aap (56). Therefore, the major debated point was the SP/AP cutoff value (between 0.32 and 0.5 in researches, reasonably no higher than 0.4) and the Asp/Aap cutoff value (53). Besides, the waveform varied a lot due to the different electrodes and stimuli (18, 56). ET-ECochG was chosen in this study due to its convenience and better acceptance in patients and inspection technicians in our center compare to the complex procedure like local anesthesia of the tympanic electrode (57). So as recommended (36), we calculated the specific diagnostic cutoff point for our laboratory. The best diagnostic cutoff point for our ET-ECochG protocol was calculated in 43 MD ears, and 80 healthy ears enrolled. The diagnostic value is 0.38 for SP/AP and 2.41 for Asp/Aap according to our results. The AUC of the ROC curve evidently represents a higher clinical significance of the index Asp/Aap and its combination with SP/AP than SP/AP only. Therefore, we recommended the ET-ECochG as a supportive examination for MD diagnosis with adding Asp/Aap as a diagnostic index, especially for outpatients.

These methodical limitations should be taken into consideration: First, the reliability of the statistical analyses would profit from a greater number of patients that were evenly distributed in all four clinical MD stages. Second, the control group varied considerably in age and gender from the investigated MD patients. Third, the MR image quality could be improved by different sequences. Fourth, there is a lack of verification through 3D quantification. For example, it had been proved that one slice of the vestibule could represent the total ELH of the vestibule, however, no literature proved that the ELH in lateral semicircular canals could represent the total ELH of semicircular and the supposed even distribution of ELH among eight parts of the semicircular canals should also be proved. Thus, further research should be conducted to assess the accuracy of the volume-referencing grading system. Furthermore, more studies with radiological and clinical follow-ups should be done to confirm the cutoff value of the point of ET-ECochG and the value of the volume-referencing grading system.

## Data Availability Statement

The raw data supporting the conclusions of this article will be made available by the authors, without undue reservation.

## Ethics Statement

The studies involving human participants were reviewed and approved by Ethics Committee of Xinhua Hospital. The patients/participants provided their written informed consent to participate in this study.

## Author Contributions

JY and MD contributed to study design, critically reviewed, and approved the final manuscript. BH and FZ contributed to the detailed study design and performed data acquisition, statistical analysis, and interpretation of results, drafting of the manuscript, and revised the manuscript. HZ contributed to the radiological supports and worked out the new grading system together with BH. XS and JC contributed to the study design, data acquisition, and statistical analysis. JC, YL, and SL contributed to the methods of statistical analysis and critically reviewed the manuscript. WW and LW contributed to ECochG data acquisition. All authors agree to be accountable for the content of the work, integrity, and accuracy of the data. All authors contributed to the article and approved the submitted version.

## Conflict of Interest

The authors declare that the research was conducted in the absence of any commercial or financial relationships that could be construed as a potential conflict of interest.
